# Ethnicity and nationality among Ethiopians in Canada’s census data: a consideration of overlapping and divergent identities

**DOI:** 10.1186/s40878-018-0075-5

**Published:** 2018-04-03

**Authors:** Daniel K. Thompson

**Affiliations:** 1 Department of Anthropology, Emory University, 1557 Dickey Drive, Atlanta, GA 30322 USA; 2 College of Social Sciences and Humanities, Jigjiga University, Jigjiga, Ethiopia

**Keywords:** Somali diaspora, Ethiopian Somalis, Canada, Ethnic identity, Oromo diaspora

## Abstract

This article addresses the intersection of ‘homeland’ politics and diaspora identities by assessing whether geopolitical changes in Ethiopia affect ethno-national identifications among Ethiopian-origin populations living abroad. Officials in Ethiopia’s largest ethnically-defined states recently began working to improve diaspora-homeland relations, historically characterised by ethnically-mobilized support for opposition and insurgency. The emergence of an ‘Ethiopian-Somali’ identity indicated in recent research, previously regarded as a contradiction in terms, is the most striking of a series of realignments between ethnicity and nationality. Such realignments reflect new orientations towards the homeland that impact diaspora engagement in politics and development. While diaspora returnees constitute a visible presence in some formerly marginalized areas of Ethiopia—including the historically disputed Somali region—large-sample data on ethnicity and nationality from Canadian censuses suggest that diaspora outreach efforts to historically marginalized groups have not (yet) effected large-scale changes in ethno-national identity, and that ongoing tensions in Ethiopia’s federal politics may have different impacts on the identities of different ethnic populations.

## Introduction

In October 2017 Toronto Restaurant joined the expanding list of diaspora returnee businesses in Jigjiga, the capital of Ethiopian Somali Regional State (ESRS). The prominence of diaspora enterprises with names like London Café, Minnesota Cosmetics, and Oslo Supermarket suggests the potential economic impacts of diaspora return to eastern Ethiopia. Diaspora return and investment in Jigjiga is part of a striking realignment between a large population of Somalis and their ‘homeland’ (by birth or ancestry) in eastern Ethiopia. A decade ago, this diaspora was perceived in terms of Somalis abroad supporting an anti-Ethiopian insurgency, the Ogaden National Liberation Front (ONLF). Today the identification ‘Ethiopian Somali’ (or Somali Ethiopian)—previously regarded as a contradiction in terms—is increasingly expressed among the region’s residents (Hagmann & Khalif, 2006), and there are indications of changes in diaspora identifications (Thompson, 2017b). The implementation of federalism in which Somalis have obtained administrative autonomy is a crucial element of this shift. Not all groups that previously supported ethnically-mobilized insurgencies, however, are happy to accept the state of affairs; in neighbouring Oromia Regional State, protests over the past two years—widely recognized as shaped by diaspora activism—have precipitated a state of crisis in the country.^1^

Ongoing power reconfigurations in Ethiopia suggest the need to examine the extent to which transformations in homelands reshape diasporic identities, with implications for political-economic developments in the Horn of Africa. Theoretically, Somali diaspora return to Ethiopia raises the point—paraphrasing Povrzanovic Frykman (2002, p. 120)—that there is no unified ‘Somali diaspora’ or ‘Ethiopian diaspora’, and that diasporas are constituted as well as internally differentiated by constellations of power, including discourses and economic processes (Basch, Glick Schiller, & Szanton Blanc, 2005; Brah, 2005, pp. 179–180). This article addresses one element of the complex picture of how ethnic and national identities are (re)constituted in diaspora by exploring whether geopolitical transformations in Ethiopia affect ethno-national identifications among people in Canada who trace their origins (by birth or ancestry) to Ethiopia, as measured in Canadian census data. The analysis begins by asking: to what extent is a shift towards Ethiopian Somali identity discernible among Somalis outside of the Horn of Africa? Officials and diaspora returnees in ESRS argue that the Ethiopian-Somali diaspora is incompletely mobilized, and the regional administration sends representatives to invite the regional diaspora home as well as devoting resources towards diaspora-oriented media programs. Adoption of Ethiopian Somali identity and enactments of it—return migration, investment, and engagement with government—could undergird political-economic transformation one of Ethiopia’s historically marginalized regions. In this regard, the article argues that Canada’s census data, while indicating the presence of non-Somalian Somalis, suggest that those identifying themselves in census enumeration as Ethiopian-Somali are fewer than could be expected. This moderates findings from a study on the US (Thompson, 2017b).

The focus on Ethiopian Somalis is an entry-point into broader considerations of the relationship between homeland politics and diaspora identities, using some of Ethiopia’s other prominent ethnic groups for comparison. While political decentralization has made some Somalis feel more Ethiopian, people in other groups such as Oromo, the largest of Ethiopia’s ethnic groups, are increasingly vocal of political discontents. Some Oromo activists in North America reject Ethiopian nationality, and policy studies in Europe suggest that ‘many Ethiopians of Oromo ethnicity see themselves as Oromo and thus not part of the Ethiopian diaspora’ (Schlenzka, 2009, p. 8). Analysis of census data suggests that Oromo are more likely than Somalis to identify themselves in census enumeration as both Oromo and Ethiopian, but the proportion of Oromo reporting multiple ethnicities has declined recently, temporally corresponding to anti-federal-government protests in Ethiopia’s Oromia Regional State. Both of these findings indicate openings for further research as well as the need for officials and development actors in Ethiopia and in Western countries to better understand the intersection between homeland politics and diaspora mobilization along identity lines.

Two considerations prompt the use of data from Canada. First, Canada’s censuses provide more detailed data on ethnicity, national origin, and generational status than other censuses in the English-speaking world. Canada’s censuses contain estimates of populations identifying with four ethnic identities commonly recognized as falling under Ethiopian nationality: Amhara, Harari, Oromo, and Tigrayan. Along with Somali, these ethnicities account for five of six ethnically-defined regional states created under Ethiopia’s 1995 federal constitution. Second, a sample of records from the ESRS Ministry of Diaspora suggests that Canadian citizens make up 10% of Somali diaspora returnees to ESRS, the fourth-largest nationality after USA, UK, and Sweden. Participant-observation and interviews during eight months of an ongoing 14-month study in Jigjiga, part of a dissertation project on diaspora return, corroborate this estimate.

This article loosely follows the approach of a study of US migration statistics which indicated the recent growth of Ethiopian-Somali identity in diaspora (Thompson, 2017b), first examining overlaps between ethnic identities and national origins in Canadian census data, then analysing data on multiple ethnic identities to discern shifts in identification among Somalis and Oromos. The first section reviews approaches to Somali and Ethiopian diasporas and outlines this study’s methodology. The second examines how the political history of the Horn of Africa shaped ethnic relations and migration trajectories, including settlement in Canada. The third and fourth sections each analyse the intersections between ethnic and national identification from a different angle. The third focuses on the mismatch between ethnic and national populations which corroborates indications in US data that a substantial portion of Somalis in diaspora are not from Somalia and likely trace their origins to Djibouti, Ethiopia and Kenya. The fourth shows that while the proportion of Somalis and ‘Ethiopian’ ethnic groups reporting multiple ethnic identities has increased, this has taken place largely among second- and third-generation groups. Oromo appear less likely in recent years to report multiple ethnic identities, suggesting that Ethiopian federalism may have distinct impacts on the expressed identities of different groups.

## Analysing Ethiopian and Somali diasporas

Academic and policy studies have explored migration trajectories of Somalis, their integration in host societies, forms of livelihood, and—more recently—the impacts of remittances and return migration on livelihoods and politics in the Horn (Abdi, 2015; Hansen, 2014; Horst, 2007; Kusow, 2006). Some studies compare patterns of Somali migration and diaspora life with a seemingly distinct Ethiopian diaspora (Danso, 2001; Grant & Thompson, 2015; Warnecke, 2010). Among academic and policy studies on Somalis in diaspora, only about 10% explicitly recognised that Somalis may originate in countries other than Somalia and sought to differentiate ethnicity from nationality (Thompson, 2017b, p. 5). Among studies relating to the Ethiopian diaspora reviewed for this study, a similar proportion mentioned Somalis from Ethiopia. It is sometimes explicitly recognised that within the category ‘Ethiopian’ there are ethnic distinctions, and that in places like Washington, D.C., Amhara—or highland *Habesha* more generally (including Amhara and Tigrayan)—tend to be numerically dominant (Chacko, 2003; Habecker, 2012).

Socio-political dynamics in the Horn and among diaspora groups suggest a more complicated picture and the need for recognizing a reality that diverges from a ‘national order of things’ (Malkki, 1992) in which the most relevant identities correspond to nation-states. Ethnicity—here taken to be a collective identity bounded by shared traits such as physical markers, cultural practices, and language (Barth, 1998)—does not necessarily correspond to political identity. Particularly in post-1945 world order, the concept of ‘nation’, on the other hand, suggests a group that may or may not map onto ethnic divisions, but is mobilized as a community seeking a set of rights, including political representation and potentially autonomy or self-determination (Kelly & Kaplan, 2001). The use of Somali as an analytic category reveals the blurry boundaries between ethnicity and nationality. As generally used in diaspora research, Somali refers to both. In practice, the first constitution of Somalia granted citizenship to all Somalis (it has remained in place since the 1960s). This complicates the picture for Somalis living outside Somalia’s boundaries, especially in neighbouring countries, since it discursively implicates Somali ethnicity with Somalian national identity and politics. Indeed, Somalis in Ethiopia fought intermittently for decades against the central government, and were frequently backed by the Somalian government in their endeavours until its 1991 collapse.

Ethnically-mobilized insurgencies fought for regional autonomy in Ethiopia leading up to the overthrow of the socialist regime in 1991, which led to Ethiopia’s major ethnic groups being explicitly recognized as separate nations in the 1995 constitution. This instituted a multi-national federal system (often referred to as ethnic federalism) which, in one sense, created the discursive possibility for a set of hyphenated ethno-nationalities, including an Ethiopian-Somali nation distinct from Somalian Somalis, considered in historical context in the following section. Discourses of nationality and the modes of belonging that they construct are complicated, especially when ethno-nationalities are seen to have varying relationships with ostensibly broader Ethiopian nationality. During informal discussions of ethnic politics in Ethiopia’s Somali Region, people who identify as Amhara suggested that ‘most Amhara do not identify themselves as Amhara, but merely as Ethiopians’ (field notes, Nov. 24, 2017). While they see this as giving nationalism precedence over ethnocentrism, people who feel marginalised in Ethiopian politics often perceive it as a claim that Amhara are the only true Ethiopians. Among Somalis in ESRS, the term ‘Ethiopian,’ when used alone, almost always refers to ‘other’ (non-Somali) Ethiopians. In this regard, the analytical separation between ‘Ethiopians’ and ‘Somalis’ in diaspora studies to some extent maps onto indigenous discursive constructs of difference, but forecloses the significance of hybrid identities that may emerge amidst geopolitical shifts.

Diaspora studies literature highlights the continuous social construction of diasporas as internally differentiated groups defined by orientation towards and interaction with a homeland that may be defined in various ways. The concept of diaspora—defined here as a migrant community oriented towards some level of socio-political involvement with the ‘home’ country, though not necessarily dedicated to return—differentiates such groups (though neither neatly nor finally) from assumptions that migrants ultimately integrate and assimilate where they settle (Brah, 2005; Quayson & Daswani, 2013). Diaspora impacts in home countries vary widely; while development institutions often highlight potential wealth redistribution through diaspora channels (Plaza & Ratha, 2011), the Ethiopian case demonstrates the importance of diaspora support for political opposition. On the other hand, it is increasingly emphasized in migration literature that relationships between diaspora and home are not straightforward: political and economic transformations in an area that is considered the homeland (by birth or ancestry) may reshape diaspora identities and modes of transnational engagement (Ali-Ali & Koser, 2002; King & Christou, 2011). The wave of return migration initiated in 2010 when Ethiopia’s Somali Regional State administration began courting Somalis abroad demonstrates how abruptly interactions can shift. Assertions in the country that diaspora mobilization is incomplete, and the fact that some diaspora groups are involved in current protests within the federal system, suggest a need to examine the extent to which Ethiopian politics have reshaped diaspora identities that might construct new modes of interaction with the homeland and with host-country governments.

Canada’s censuses provide more useful data through which to analyse such shifts than data from many other Western censuses, first in that they include detailed data on ethnicity and generational status; and second in that the Beyond 20/20 software from the Stat Canada website enables a multi-dimensional analysis of variables. Data utilised in this study are drawn from 5-year Canada census data from 1996 to 2016. (Tables utilised are listed at the end of this article.) Tables on immigration by country of birth and on ethnic populations were exported from Beyond 20/20 as comma-separated value (csv) files and imported into R Studio software for analysis and visualization. Data are interpreted in light of insights from an ongoing field study of diaspora return in eastern Ethiopia (information at the time of writing is based on the first eight months of a 14-month fieldwork period).

## Politics in the Horn and the changing diaspora scene

**Fig. 1:**
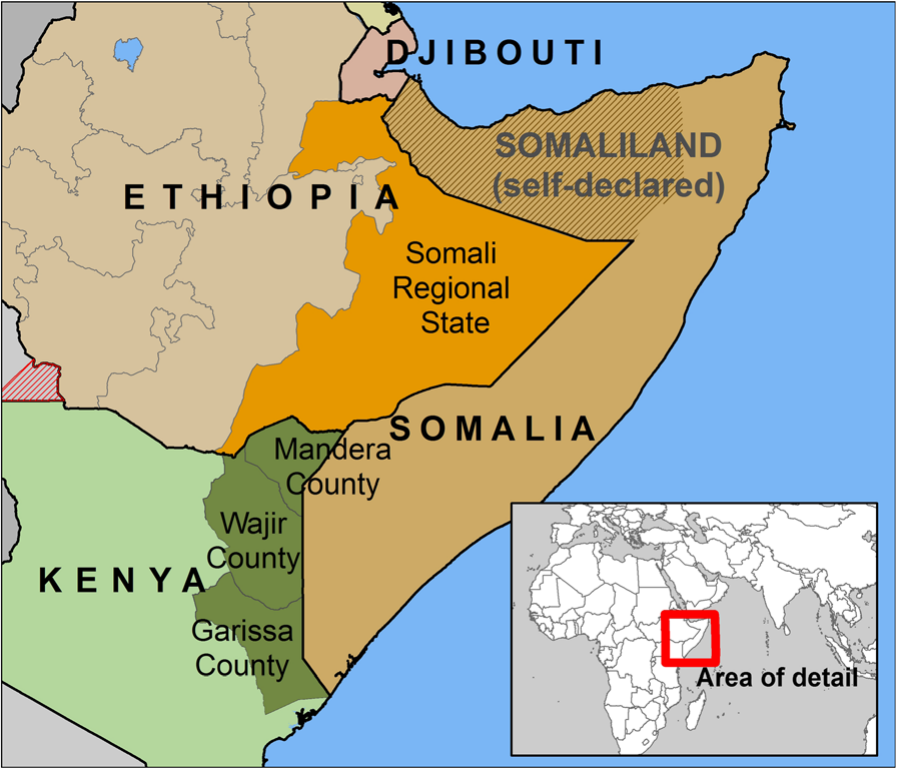
The Horn of Africa, showing administrative areas of Kenya and Ethiopia that are predominantly inhabited by Somalis

Somalia’s protracted ‘statelessness’ since 1991 generated a massive diaspora. UN estimates place the number of Somalians living outside of Somalia as of 2010 at nearly 1.6 million, equivalent to almost 17% of Somalia’s estimated resident population (UNDESA 2015). Eritrean, Ethiopian, and Kenyan populations abroad are also substantial, though much smaller proportional to populations remaining in each country. The bulk of Somalians, as with other expatriates from countries in the Horn of Africa, live within the region: 5-year UN migration estimates place the number of Somalians living in Kenya and Ethiopia as of 2015 at over 900,000 (UNDESA 2015). Along with the large Somalian populations living (many as refugees) in Kenya and Ethiopia, there are also several million Somalis indigenous to the eastern parts of these countries. Kenya’s most recent census (2009) estimates 1.2 million inhabitants in the predominantly-Somali counties of Garissa, Mandera, and Wajir.^2^ Ethiopia’s (2007) places the population of Ethiopia’s Somali Regional State at nearly 3.5 million (Fig. 1).

The histories of these regions generated tensions between Somali ethnic identification and Somalian nationality. On the eve of Kenya’s independence, British administrators held a referendum in what was then Kenya’s Northern Frontier District. Despite their finding that a majority of Somalis favoured union with Somalia, the British government granted Kenya independence with its territoriality intact (Samatar, 2013, p. 158). From 1963 to 1967 the ‘Shifta’ insurgency made Somalis suspect as citizens of Kenya; amidst al-Shabaab attacks in Kenya^3^ and a history of trans-border movement, elements of suspicion remain today. In Ethiopia, likewise, Somali-inhabited land that became Ethiopian territory under an 1897 treaty was effectively unified with British Somaliland and former Italian Somaliland under British administration during the 1940s but returned to Ethiopian sovereignty in 1954. The 1960s witnessed Somali secessionist insurgency along Ethiopia’s eastern border, and in 1977 Somalia’s government support for secessionists turned into full-scale invasion in an attempt to unify Somali-inhabited territory. Somalia’s president, Siyad Barre, also sought to mobilise other marginalised groups in Ethiopia—including Oromos—to back Somalia in the conflict.

Colonial and postcolonial political developments in the Horn shaped migration patterns. Somali men, originating in a culture centred on nomadic pastoralism, were already migrating to and from Cardiff, Bombay, Mecca, and Aden before 1900. Somali seamen who had joined Britain’s navy or merchant marine were prominent participants in famous riots of ‘coloured seamen’ in British ports in 1919 and 1930 (Byrne, 1977). Not only Somalis, but Oromos (who comprise a large indigenous Kenyan population as well) and other groups from the Horn had diasporic elements by the early twentieth century. Oromos, for example, have over the past two centuries constituted significant populations in southwest Asia (Bulcha, 2002).

Migration within, and emigration away from, the Horn accelerated amidst conflict from the 1970s onwards. The 1977 Ethio-Somali war drove migration to North America, followed by continued arrivals during political turmoil in Ethiopia and Somalia throughout the 1980s–1990s. Oromos living alongside Somalis in eastern Ethiopia also mobilized against the Ethiopian government, and a stream of Oromos fled abroad, though less has been written of this migration than of the Somali case. Insurgency and government repression led to mass flight from other parts of Ethiopia. Two decades of conflict led up to Eritrea’s independence from Ethiopia in 1993; border conflict in 1998–99 caused more emigration from Ethiopia-Eritrea border areas, a region inhabited largely by Tigrayans.

Yet the well-known narrative of emigration must also take account of political shifts that have changed orientations, networks of financial support and investment, and migration *within* and *towards* the region, not only away from it. When Somalia’s government collapsed, the northern part of the country declared independence as the Republic of Somaliland, and since the 1990s has had a functioning democratic government despite its lack of international recognition. By the early 2000s, return migration to the self-declared Republic of Somaliland facilitated economic growth and brought people with experience in Western democracies ‘home’ to participate in state-building (Hammond, 2015; Hansen, 2014). February 2017 Elections in Somalia saw Moḥamed ʿAbdullahi ‘Farmajo’ Moḥamed, a dual citizen of the US and Somalia, chosen as president. While the Horn became famous during the 1990s for its emigrants, new patterns of immigration and orientations of diaspora populations toward their ‘homelands’ are crucial to understand for their potential contributions to political and economic change. Diaspora populations’ ideas about who they are and where they belong—including self-identification with ethnic and national categories—reflect important elements of orientation towards and potential engagement with regions of origin.

### Ethnic and national identification in diaspora

There are indications that political transformations in the Horn have brought about shifts in political identification both in the region and farther afield. The adoption of ‘Ethiopian-Somali’ identity is probably the starkest of these transformations, given that these two categories appeared so sharply at odds in political discourse before the 1990s. However, the feeling of identities at odds with Ethiopia is not relegated to Somalis who ostensibly have a second citizenship in Somalia. Outspoken Oromo nationalists in diaspora have argued that ‘Ethiopia is a symbol of racial/ethnonational oppression and exploitation’ and Oromos ‘never assumed an Ethiopian identity for themselves’ (Jalata, 2002, pp. 135-136). Protestors in Ethiopia’s Oromia Regional State are currently pushing not for separatism, but for democracy and de-ethnicization of Ethiopian citizenship, while rejecting symbols of past dominance by minority groups, such as Amharic place-names given to Oromo-inhabited areas.

When people from groups structured by such complex relations between ethnic and national identities migrate abroad—often as refugees—they may claim (or have forced on them) the national identity of a country to which they have never been or with which their connections are not immediate or personally felt. For example, Ethiopian and Kenyan Somalis as well as Oromos—some who had fled to Somalia during the conflicts of the 1960s and 1970s, but others who had never set foot in Somalia’s territory—sometimes seek and obtain recognition as refugees from Somalia in refugee-receiving countries (Thompson, 2017a). This has been recognised by governments in refugee-receiving countries, giving rise to intense vetting procedures to determine if Somalis are really Somalian (Wettergren & Wikström, 2014). Such procedures assume that the situation in the cookie-cutter geopolitical unit known as Somalia is more worthy of escaping than the situation immediately across the border—which may or may not be the case, since borders seldom neatly contain humanitarian crises.

A recent study of data from the US American Communities Survey finds several revealing trends relating to ethnicity and nationality. First, the study finds that the population of Somalis in the US is significantly higher than the population of people born in Somalia, and that estimates of the population of second-generation ethnic Somalis (those born in the US) cannot account for this. Second, it finds an increase from 2009 to 2015 in Somalis reporting multiple ancestral identities in the same locations as an increase in reports of Ethiopian multiple-ancestry, suggesting an emerging Ethiopian-Somali identity (Thompson, 2017b). While the populations and sample sizes (based on census geographical units) in Canada’s census data are smaller than those in the US, a number of comparable variables and some additional relevant variables not included in US surveys—including more specific ethnic identification and estimates of first, second, and third generations—offer an opportunity to test these findings and offer a comparative analysis.

### Migration from the Horn to Canada

**Fig. 2:**
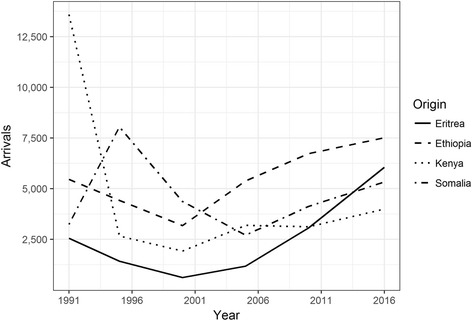
Estimated migration from countries in the Horn of Africa to Canada for 5-year periods ending 1995–2016 (1991 includes all migrant arrivals prior to 1991)

**Fig. 3:**
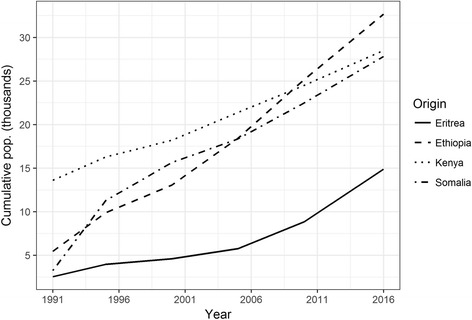
Estimated cumulative migrant populations from countries in the Horn of Africa, 1991–2016

Like the US and several European countries, Canada hosts a significant population from the Horn—largely refugees—who began to arrive in large numbers during the early 1990s. Between 1991 and 1995, the largest number of arrivals from the Horn were people born in Somalia, followed by Ethiopia-born, Kenya-born, and Eritrea-born (note that Eritrea was officially part of Ethiopia from 1953 until 1993, complicating categorizations). By 2010 Ethiopian-born made up the greatest foreign-born population in Canada from among the four countries (Figs. 2 and 3). Not all people came directly from the Horn to the receiving country. For example, hundreds of Somalis in South Africa, facing xenophobic violence, obtained refugee resettlement in North American cities including Minneapolis, Vancouver, and Toronto (personal communication with resettled families, 2010–2016). Moreover, as Danso (2001) observes, during the 1990s most Ethiopians were selected outside of Canada and arrived with permanent residence status while most Somalis came to Canada as refugee claimants.^4^

**Fig. 4:**
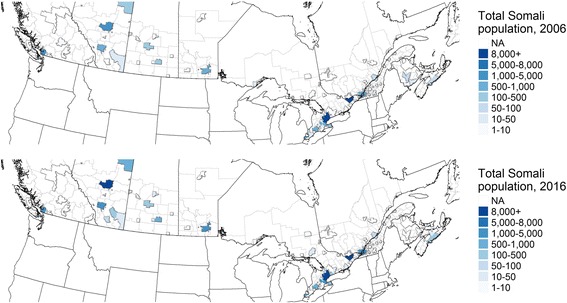
Somali populations in Canada's Census Metropolitan Areas (CMAs), 2006 and 2016

**Fig. 5:**
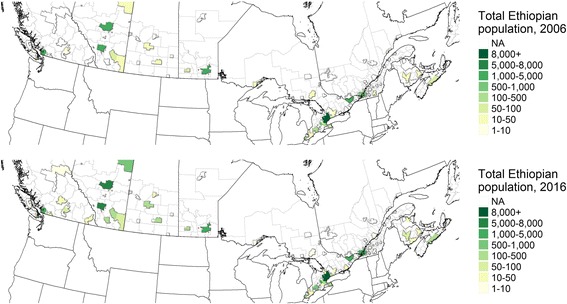
Ethiopian populations in Canada's CMAs, 2006 and 2016

Populations from the Horn tended to concentrate in a few Canadian metropolitan areas. As of 2016, Toronto was the predominant location of settlement for Somalis, followed by Ottawa, Edmonton, Calgary, Vancouver and Winnipeg. The order for Ethiopians is the same. These distributions are essentially steady from 2006 to 2016, although populations of each group in these core locations increased (Figs. 4 and 5).

**Fig. 6:**
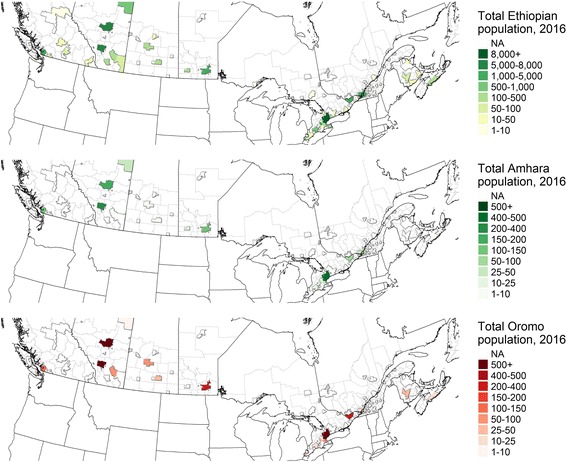
Ethiopian (top), Amhara (center) and Oromo (bottom) populations in Canada, 2016

Estimates suggest many fewer people reporting major Ethiopian-origin ethnic identities—Amhara (1530 in 2016, including estimates of single and multiple ethnicities), Harari (660), Oromo (3350), and Tigrayan (2155)—as reporting their ethnicity simply as ‘Ethiopian’ (44,060). Whatever the felt connections between national and ethnic identity, the presence of statistics on ethnic populations provides indications of settlement patterns among co-nationals from different groups. In other settings, Oromo live alongside Somalis in significant numbers (Carrier & Lochery, 2013; Thompson, 2017a). While Oromo are often invisible in Somali diaspora studies, patterns of co-residence may both reflect affinities between groups and facilitate interactions that shape diaspora politics. Amidst ongoing political jostling in Ethiopia, for example, certain Somali clans in diaspora aligned with Oromo protestors against the ESRS administration, prompting meetings in Minneapolis.^5^ In Canada, data on ethnicity from 2016 include census metropolitan areas (CMAs), which are too large to discern finer-grained settlement patterns. Even in 2006 data on ethnicity for Canada’s census divisions (CDs) and subdivisions (CSDs), populations of Ethiopians (including sub-groups) and Somalis appear concentrated together: the estimated ethnic populations are highly correlated. While the small sample size necessitates caution regarding conclusions, the correlation between Somali and Oromo populations in CDs and CSDs appears higher than that between Amhara and Oromo or Amhara and Somali (Table 1). The 2016 CMA data show a number of metropolitan areas in which Amhara and Oromo do not overlap, or where the bulk of the Ethiopian population appears to be accounted for by one or another of the groups (Fig. 6).

**Table 1:** Co-residence of selected ethnic groups in census divisions and census subdivisions, 2006 census

Ethnic groups	Correlation coefficient (R)	Number of CSDs with populations of both groups
Amhara and Oromo	0.847	16
Amhara and Somali	0.817	17
Amhara and Ethiopian	0.940	18
Oromo and Ethiopian	0.957	18
Somali and Oromo	0.905	18
Somali and Ethiopian	0.931	41

## Ethnicity and national origins

If Somalis tracing their origins to Ethiopia and Kenya make up substantial populations in Canada, and if they report these origins to census enumerators, then Somalis should consistently outnumber the Somalia-born population. Calculating the proportion of reported ancestry to country of birth in the US indicates that likely between 16 and 25% of the US Somali population is not Somalia-born (Thompson, 2017b, p. 17). In US data, the proportion of ancestry to birth estimated for Somalis in 2015 was 1.424, compared to 1.331 for Ethiopian ancestry to Ethiopian birth. In a selected subset of counties, this proportion rose to 1.835 for Somalis, compared to 1.364 for Ethiopians. Given the longer history of Ethiopian migration to the US, it is expected that the population of second-generation Ethiopians in the US is higher than that of Somalis—and higher than that of Ethiopians in Canada as well. The proportion of Somalis to populations reporting birth in Somalia across Canada’s CMA data is similar to that found for US counties, with a consistent proportion of Somali ethnicity to Somalian birth of over 1.4 from 1996 to 2016, compared to a proportion of Ethiopian ethnicity to Ethiopian birth of less than 1.2 (the 1996 proportion for Ethiopians is higher and difficult to interpret, but proportions are consistent from 2001 to 2016) (Table 2). The proportion of Somali ethnicity to Somali birth across Canada’s CMAs is significantly higher than the corresponding Ethiopian proportion (*p* < 0.05 for 2001, *p* < 0.001 for subsequent years). As with US data, Kenya-born people appear less likely than Somalis or Ethiopians to report their ethnicity as ‘Kenyan.’

**Table 2:** Proportion of ethnicity to birth for groups from the Horn of Africa, 1996–2016 censuses

Country of ancestry/birth	Year	N (CSDs or CMAs)	Mean proportion, ethnicity to birth	Median proportion
Ethiopia	1996	25	1.776	1.702
	2001	31	1.029	1
	2006	43	1.1017	1
	2011	21	1.198	1.168
	2016	66	1.133	1.119
Eritrea	1996	NA	NA	NA
	2001	25	1.314	1.286
	2006	25	1.533	1.649
	2011	NA	NA	NA
	2016	45	1.43	1.5
Kenya	1996	NA	NA	NA
	2001	60	0.203	0.079
	2006	64	0.222	0.049
	2011	26	0.546	0.521
	2016	72	0.435	0.327
Somali	1996	26	1.545	1.574
	2001	27	1.375	1.409
	2006	36	1.628	1.802
	2011	NA	NA	NA
	2016	39	1.986	2.047

Considering a subset of CMAs with estimated Somali-ancestry and Ethiopia-born populations both of at least 50, the difference in proportions of ancestry to birth in 2016 data is striking. Within this set of 31 CMAs, the number of Somalis is more than twice the Somalia-born population. The corresponding proportion for Ethiopians is 1.25 (Table 3).

**Table 3:** Proportion of ethnicity to birth for selected CMAs, 2016 census

Country of ancestry/birth	Year	N	Mean proportion, ethnicity to birth	Median proportion
CMAs with ethnic Somali and Ethiopia-born population both at least 50				
Somalia	2016	31	2.246	2.148
Ethiopia	2016	31	1.262	1.238
CMAs with ethnic Somali and Kenya-born population both at least 50				
Somalia	2016	29	2.293	2.148
Kenya	2016	29	0.467	0.410

Unlike ACS data from the US, Canada’s data include estimates of first-, second-, and third-generation populations for each of these groups. For 2006, data on ethnicity and generational status available on Canada’s census website at the time of writing were not based on the same geography as data on country of birth. Therefore, the percentage that the first generation comprised for each ethnic group across Canada as a whole was used to estimate the proportion of first-generation Somalis to individuals born in Somalia. Estimates of second and third generations for each group grew significantly from 2006 to 2016; nevertheless, the number of first-generation Somalis continued to outnumber the Somalia-born population at a ratio that corroborates estimates in the US that up to 20–25% of ethnic Somalis in diaspora trace their origins to Ethiopia or Kenya (and a smaller number to Djibouti) (Table 4). The difference in means between the proportion of Somalis and the proportion of Ethiopians is significant for both years (*p* = 0.0131 for 2016; *p* < 0.001 for 2006).

**Table 4:** Median and mean proportions of ethnicity to birth for first-generation Somalis and Ethiopians, 2006 and 2016 censuses

Proportion	Geography	Year	N	Med	Mean
Somali ethnicity/Somalian birth	Canada	2006	1	1.440	1.440
	Canada	2016	1	1.282	1.282
	CDs & CSDs	2006	36	1.384	1.250
	CMAs	2016	23	1.274	1.132
Ethiopian ethnicity/Ethiopian birth	Canada	2006	1	0.924	0.924
	Canada	2016	1	0.860	0.860
	CDs & CSDs	2006	43	0.806	0.820
	CMAs	2016	44	0.816	0.730

**Fig. 7:**
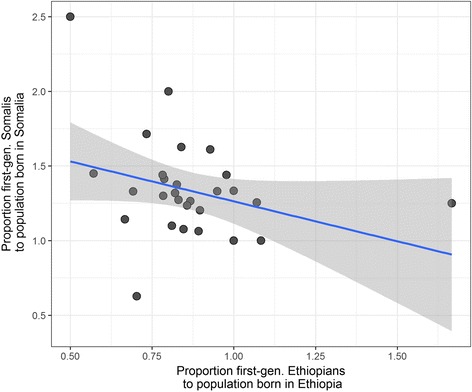
Proportion of first-generation Somalis to population born in Somalia, plotted against proportion of first-generation Ethiopians to population born in Ethiopia for CMAs with populations of both groups. Blue line indicates linear regression trend and grey area is 95% confidence interval for the regression line

One indication that Ethiopian Somalis account for some of this discrepancy between ethnicity and national origins is that a negative correlation exists between the proportion of Somalis to Somalia-born and the proportion of Ethiopians to Ethiopia-born. If there are areas with concentrated populations of Ethiopian-born Somalis, these areas should have a relatively high proportion of Somali ethnicity to birth in Somalia combined with a low proportion of Ethiopian ethnicity to Ethiopian birth. The trend among the 29 CMAs that had populations of Somalis and Ethiopians in 2016 is a weak negative correlation (R^2^ = 0.103) (Fig. 7). This is again similar to findings among U.S. counties. The fact that these data include only first-generation immigrants, whereas the ACS data do not, further supports the thesis.

The indication that one-quarter of Somalis in Canada are not from Somalia has several implications. First, it is not to be assumed that these Somalis are illegal immigrants posing as Somalian refugees. As indicated above, a huge number of Ethiopian-born Somalis *are*, in practice, Somali refugees, having taken up their Somalian citizenship and lived in Somalia from the 1970s until 1991 (field notes and interviews, January 2017–January 2018). Second, even if some are not from Somalia, the insecure environment in eastern Ethiopia and Kenya over the past two decades has often been little different from the ‘statelessness’ of Somalia, raising a difficult conceptual space between regimes of refugee law that are based on nation-state classifications and the ethical impetus behind such law, which suggests that anyone in fear for their life should be allowed to move elsewhere. The contrast between ‘stateless’ Somalia and the apparent presence of government in neighbouring countries is a stereotypical image, undergirding an assumption that governance and rights are exercised equally across territory, and contributing to the reproduction of a national order of things in migration policy (Gill, 2010).

Third, the indication of significant non-Somalian Somali populations—and the ways that their identification with Somalian nationality has been tied to suspicion and marginalization in Kenya and Ethiopia—is an example that should challenge researchers to carefully conceptualise study populations and the processes shaping their self-identifications.

## Single and multiple ethnic origins

In the US and Canada, surveys on ethnicity are categorised by the total number of people reporting an ethnicity, the number of people reporting one ethnicity alone (single ethnicity), and the number of people reporting an ethnicity in conjunction with others (multiple ethnicity), though these other ethnicities are not specified in disaggregated data. In the US, it appears that from 2009 to 2015 the percentage of Somalis reporting multiple ethnicity more than tripled in both a broad selection of counties with Somali and Ethiopian populations and a selected sample of counties with Somali and Ethiopian populations of at least 1000 (Thompson, 2017b, pp. 20-21). This increase corresponds with diaspora return to Ethiopia’s Somali Regional State during the current administration. The short time during which this dramatic change took place indicates it probably results from a shift in expressed identity among Somalis tracing their origins to Ethiopia.

**Fig. 8:**
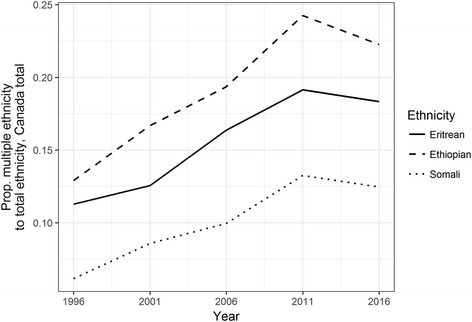
Proportion of people reporting Eritrean, Ethiopian, and Somali ethnicity who reported multiple rather than single ethnic identity, 1996–2016

In Canada, data available on multiple ethnicity is less fine-grained (both spatially and temporally) than the US ACS data, and data on multiple ethnicity from 1996 to 2016 available online do not correspond internally in terms of geographical units. Nevertheless, enough information is available for a general analysis of trends comparable to those in the US. Similar to trends in the US, the proportion of Ethiopians and Somalis (as well as Eritreans) reporting multiple ethnicity has increased over time (Fig. 8). While the proportion of Somalis reporting multiple ethnicity is similar to that in the US (between 0.5 and 0.15), the proportion of Ethiopians reporting multiple ethnicities is higher, perhaps reflecting Canada’s inclusion of more ethnic categories in censuses. Neither the increase in the proportion of Ethiopian multiple-ethnicity populations nor that among Somalis is as great as that for the US data (Thompson, 2017b), especially when focusing on the past ten years that would roughly correspond to the US 2009–2015 ACS estimates. From 2006, the percentage of Somalis reporting multiple ethnicities increased from 10 to 12.5%, while the corresponding percentage of Ethiopians also rose by about 3 percentage points.

Most of this change, among both Somali and Ethiopian groups, can be accounted for by second and third generations being more likely to report multiple ethnicities. Summary data for Canada indicates that second and third generations tend to be twice as likely to report multiple ethnicities as the first generation. Across all generations, the proportion of Somalis reporting multiple ethnicities is lower than that of other groups under consideration. Only 8.1% of first-generation Somalis in 2016 reported multiple ethnic identities, compared with 16% of Ethiopians. In the second generation, almost 18% of Somalis reported multiple ethnic identities—perhaps adopting Somali-Canadian identity (which I have heard employed by Somalis who grew up in Canada) (Table 5). Regarding the increase in multiple-ethnicity populations, the main driver of the change appears to be the increased representation of second- and third-generation Somalis in the data from 6% in 2006 to 42% in 2016.

**Table 5:** Proportions of generational groups reporting multiple ethnic identities in 2016 census

Ethnic origin	Total prop. reporting multiple	Prop. 1st gen. reporting multiple	Prop. 2nd gen.	Prop. 3rd gen.
Total (all ethnicities)	0.411	0.178	0.453	0.493
Southern and Eastern Africa	0.333	0.236	0.451	0.936
Amhara	0.536	0.489	0.676	1.00
Eritrean	0.183	0.136	0.304	0.947
Ethiopian	0.223	0.160	0.336	0.798
Kenyan	0.501	0.355	0.764	0.958
Oromo	0.282	0.243	0.353	1.00
Somali	0.125	0.081	0.179	0.662

Even in a subset of CMAs with Somali and Ethiopian-born populations of at least 50, a change in Somalis reporting multiple ethnicities is not evident. Among 17 CMAs meeting that specification in 2006, the mean proportion of Ethiopian multiple ethnicity was 0.201 (median 0.188); the mean for Somalis was 0.114 (median 0.127). Among 23 CMAs meeting the specification in 2016, the mean proportion of Ethiopian multiple ethnicity was 0.148 (median 0.221); the mean for Somalis was 0.106 (median 0.127). Based on averages across CMAs as well as within this subset of CMAs, data on multiple ethnicities from 2006 to 2016 do not corroborate findings from the US regarding the increase in Somalis reporting multiple ethnicities. It should be recognised that the smaller number of geographical statistical units included in Canada’s data, different sample populations and the high concentration of study populations in a few metropolitan areas might confound some of these trends.

**Fig. 9:**
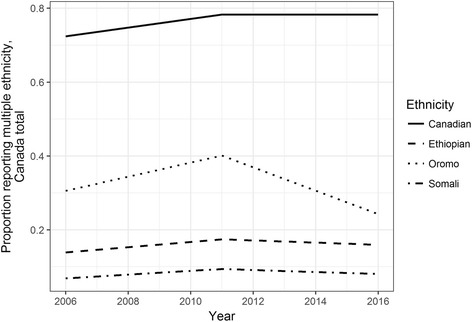
Proportion of first-generation populations who reported multiple ethnicity, 2006–2016

Calculating the proportion of first-generation Somalis and Ethiopians reporting multiple ethnicities from 2006 to 2016 reveals a relatively flat trend. The percentage of first-generation Somalis reporting multiple ethnicities hovered below 10%, and the percentage of Ethiopians likewise remained relatively stable over the period, at below 20% (Fig. 9). Among other groups that correspond to a broader Ethiopian identity, the percentage of Oromo reporting multiple ethnic identities appears to have fallen between 2011 and 2016. These trends suggest that federal politics in Ethiopia may not have encouraged the widespread adoption of a hybrid Ethiopian identity in diaspora, raising more questions about how federal politics are shaping overlaps and disjunctures between Ethiopian identity and ethno-national categories both within the country and among diaspora groups.

### Oromo multiple ethnicity

While a full examination of diasporic politics among Oromos is beyond the scope of this article, any suggestion of how current political transformations in the Horn may affect diaspora groups would be incomplete without a comment on the changing transnational Oromo political scene, if only to suggest some directions for future research. Data displayed in Fig. 8 above suggest a reversal of the upward trend in multiple-ethnicity populations between 2011 and 2016. Groups likely to report multiple ethnicities may be concentrated in particular CMAs, making broader-scale analysis difficult. However, the trend deserves attention because of its temporal correspondence with political shifts in Ethiopia.

Shifts in political mobilization among Ethiopia’s Oromo population have been increasingly visible since 2015. Amidst the implementation of federal decentralization in Ethiopia from the early 1990s onwards, Oromo politicians and nationalists have decried continuing practical centralization of power under Tigrayan leadership (Jalata, 2001). Over the past two years, these discontents have fed large-scale protests in Ethiopia’s Oromia Regional State. It is ‘common knowledge’ in Ethiopia that these protests are supported by diaspora political leaders, especially diaspora activists connected to media outlets such as the Oromia Media Network. Quantitative data are scant, but an analysis of multiple ethnicities leads to tentative observations.

**Fig. 10:**
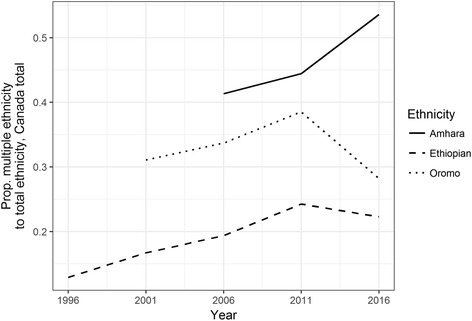
Proportion of selected groups who reported multiple ethnic identities, 1996–2016

Canadian census data from 2001 onwards include Oromo—and data from 2006 onwards include Amhara, Harari, and Tigrayan groups as well. The proportion of Oromos reporting multiple ethnicities was higher than that of Ethiopians or Somalis from 2006 onwards. (It is likely that in census enumeration, many Oromos simply report themselves as Ethiopian, and many of those who do report being Oromo probably report it as an addition to Ethiopian.) As is the case with Eritrean, Ethiopian, and Somali populations as recorded in the ethnicity data, the percentage of Oromos reporting multiple ethnicities increased from 2006 until 2011. After this, the percentage declined, so that the estimated proportion of multiple-ethnicity Oromo in 2016 is lower than it was in 2001 (Fig. 10). This decline corresponds temporally to widespread mobilization in Ethiopia and among members of the Oromo diaspora. It is noticeable in Table 5 that the percentage of Oromo reporting multiple ethnicities increased less dramatically between the first and second generation than the corresponding percentage for other ethnicities (except Amhara). More research is needed to verify these trends, and a fuller comparative analysis of how different Ethiopian ethnicities are mobilized at home and in diaspora is important but beyond the scope of the current study. What is notable in the data is that the proportion of some other Ethiopian groups (Amhara and Harari) reporting multiple ethnicity increased during the same period (Tigrayan multiple ethnicity, however, declined). Ethiopian federalism, redistributing access to power and resources in the country along ethno-national lines, has had different impacts on different ethno-national groups within the country and in diaspora. Oromo political unity fostered by the federal system, combined with widespread support in Ethiopia for Oromia Regional State’s leadership in their calls for reform, has given Oromo in diaspora a strong platform on which to engage foreign governments. Oromo organizations in the US pushed for resolutions currently under consideration in the US Congress regarding democracy and human rights in Ethiopia, suggesting how power reconfigurations in Ethiopia might connect to new forms of transnational political engagement.

### Ethiopian Somalis, multiple ethnicity and diaspora return

Even if Canada’s census reports do not confirm a broad identity shift among Somalis tracing their origins or ancestry to Ethiopia, there are some location-specific indications of both the presence of Ethiopian Somalis in Canada and their engagement as diaspora with ESRS. Among Canada’s metropolitan areas, Toronto hosts the largest population from the Horn of Africa. From 2011 to 2016, the estimated population of first-generation Somalis in Toronto grew by 2000, or 17.4%. The corresponding increase in the single-ethnicity Somali population was 1875 (17.6%), while the increase in the (first-generation) multiple-ethnicity Somali population was 140 (14.3%). Meanwhile, the estimated population of Oromos grew by 45, or 10.6%. The corresponding increase in the single-ethnicity first-generation Oromo population was 100 (39.2%), while the multiple-ethnicity first-generation Oromo population decreased by 15 (8.8%). The estimated population of Ethiopians grew by 2000 (24.4%), single by 1745 (25.8%), and multiple by 250 (17.2%). These trends reflect the broader patterns discernible in the data, though given the small sample size there is need for substantiation in future research.

**Fig. 11:**
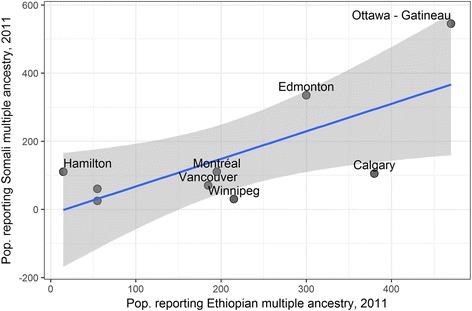
Population reporting Somali multiple ethnicity plotted against population reporting Ethiopian multiple ethnicity in 2011, selected metropolitan areas (excluding Toronto). Blue line indicates linear regression fit, and grey area is 95% confidence interval for the regression line

**Fig. 12:**
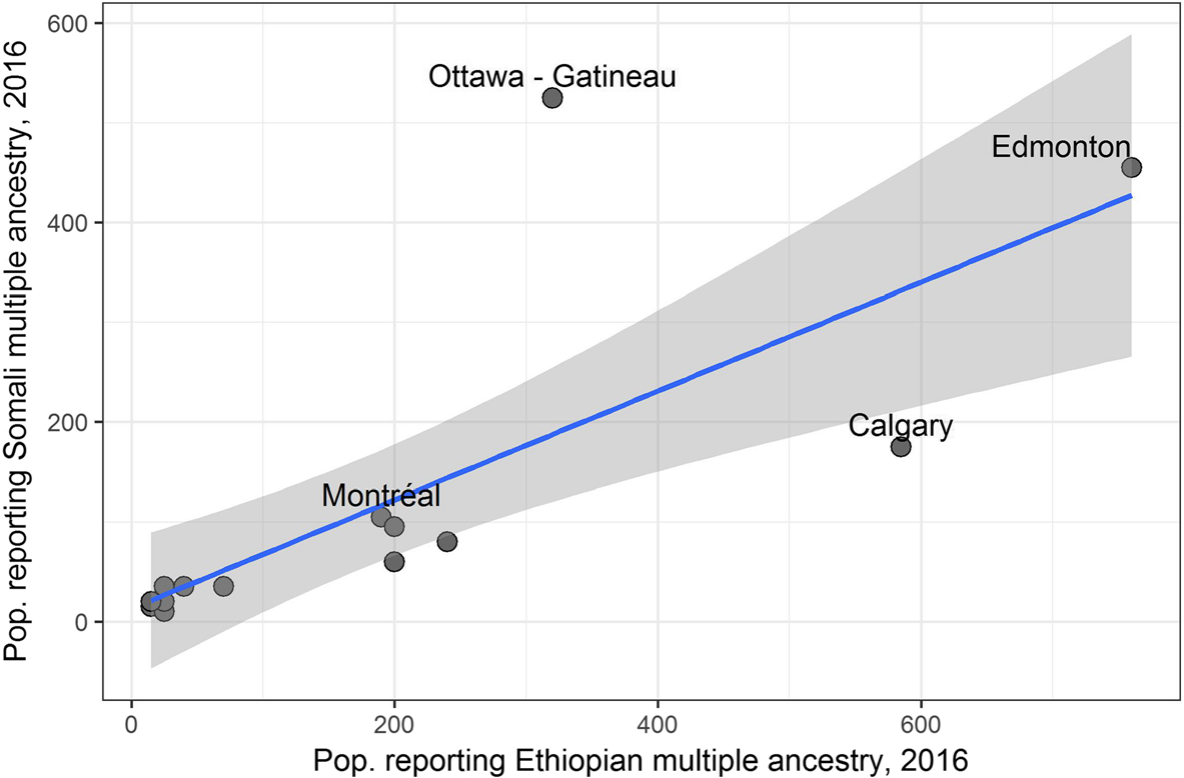
Population reporting Somali multiple ethnicity plotted against population reporting Ethiopian ethnicity in 2016, selected metropolitan areas (excluding Toronto)

Outside of Toronto, the metropolitan areas with the largest first-generation populations reporting multiple Ethiopian ethnicity and multiple Somali ethnicity are Ottawa, Edmonton, Calgary, Montreal, Vancouver, Winnipeg, and Hamilton. Among these, Edmonton is notable for the growth of its estimated multiple-ethnicity populations from 2011 to 2015 (Figs. 11 and 12). Estimates indicate that both the Ethiopian and Somali populations in Edmonton grew dramatically from 2011 to 2016. The estimated first-generation population reporting multiple ethnicity-Ethiopian more than doubled from 300 to 760 (the single-ethnicity first-generation Ethiopian population increased by 70.3%), while the first-generation population reporting multiple ethnicity-Somali grew by 35.8%, from 335 to 455. Beyond this, however, Edmonton and other locations with relatively high multiple-Ethiopian and multiple-Somali ethnicity in the census data correspond to data obtained from the ESRS Ministry of Diaspora.

A subset of diaspora records provided by the ESRS Ministry of Diaspora in 2016 lists 595 diaspora returnees who registered in 2014–2015.^6^ Returnees from Canada make up the fourth-largest group of registered returnees in this data. Of 595 cases (50 from Canada), telephone country codes were available for 339, and of these sub-country telephone area codes were listed for 237 (27 from Canada). Canada’s phone system has a combination of location-specific area codes and newer (mostly mobile phone) area codes that cover broader regions. The largest number of Canadian telephone area codes registered were Alberta numbers (12 unique numbers, including two identifiably from Edmonton). Alberta is followed by Ontario (8 unique, including three from Toronto), and area codes for Winnipeg (Manitoba), British Columbia, Sasketchewan, and Ottowa each had one corresponding registered returnee. People move, and it cannot be assumed that cell phone area codes correspond to the present residence of Ethiopian Somalis in Canada. Nevertheless, the phone numbers suggest where these individuals may have lived previously, and the match of these locations with census data showing populations reporting Somali multiple ethnicity and Ethiopian multiple ethnicity supports the thesis that Ethiopian Somalis are identifiable by multiple ethnicity data from Canadian censuses. Even if the adoption of an Ethiopian-Somali identity is not widespread across the whole population, a significant number of individuals, especially in Edmonton and Toronto, do appear to be both engaging with ESRS—including making return visits and limited investment—and identifying themselves as Ethiopian Somalis. The question remains as to whether these trends are the beginning of a wider shift.

## Conclusion

According to accounts of diaspora returnees in ESRS, the possibility of being Ethiopian *and* Somali emerged mainly in the context of the federal system, and actual adoption and enactment of this identity among the Somali diaspora began to accelerate very recently—around 2010, when ESRS began diaspora outreach efforts. While the assertion that ethnic and national identifications are malleable categories is nothing new to social science, the potential changes explored here—a shift among Somalis towards Ethiopian-Somali identity, and a shift among Oromo away from Ethiopian identity—have three implications for scholarship and policy.

First, while theories of diaspora for have some time emphasized internal differentiation within ethnically- or nationally-defined diasporas, the Somali diaspora, among the ‘poster-children’ of forced migration, is mainly represented in popular discourse and analysed in academic studies as a relatively unitary group, defined in terms of refugeehood and displacement from the ‘failed state’ of Somalia. While Somalis from Ethiopia, Kenya, and Djibouti often have affective ties to Somalia, and perhaps even close relations living there, the fact that about one-quarter of the Somali diaspora can identify its homeland, in terms of birth or ancestry, outside of Somalia constitutes a significant oversight in analyses to date. The emergence, however nascent, of an Ethiopian-Somali identity is a significant trend, and yet in the decade following Hagmann and Khalif’s (2006) study of the possibilities for this identity amidst the changing politics in eastern Ethiopia, little more has been written on the topic, much less in relation to diaspora studies. This oversight is paralleled by the significant absence of studies focusing on Oromo migration and diaspora formations: a search of articles published in top migration journals in the past two decades reveals little scholarship focusing on this large and internally diverse identity group.

Second, diaspora mobilization and identity shifts have major implications in the realm of politics and international development. If some Somalis are taking up Ethiopian identity while some Oromos are tending to reject Ethiopian identity in connection with Ethiopia’s federal politics, these trends are likely to shape political stability as well as development finance in the Horn. The fact that such shifts in identity are not final or irreversible has both reassuring and worrying implications: If Somalis can become ‘more Ethiopian’ through political changes in the homeland, so can Oromos; on the other hand, the fragility of the shift towards Ethiopian-Somali identity is suggested by its short historical depth and its mobilization of what appears to be a relatively small portion of the potential Ethiopian-Somalis among the broader Somali diaspora. In a short time, diaspora engagement in ESRS has connected with government initiatives to promote economic development. The stability of Ethiopia’s Somali Region is crucial for efforts at state construction in Somalia itself, since it closes options for insurgent groups to escape across borders and also mediates the destabilizing role of Ethiopian intervention in Somalian politics—thus the role of diaspora is important not only for Ethiopia, but for regional politics in the Horn. Initiatives such as current Oromo diaspora pressure on Western governments are also important to follow for their immediate impacts on Ethiopian politics and potential long-term effects. Given that diaspora groups play important roles—whether it is supporting development or supporting insurgency—policymakers should adjust to the mutual shaping of diaspora and homeland political mobilization.

Third, in relation to broader issues of migration and diaspora research, given the speed of travel and immediate nature of communications that facilitate relationships and mobilization of groups in various places, a view towards multi-sited research is essential. Even if time and resources constrain conducting fieldwork in multiple locations, at least a consideration of the multi-sited connections and potential futures of diaspora groups is crucial. It seems challenging enough to take into account the complicated contexts of reception in migration research and to study diaspora populations outside of their ‘homelands,’ and in-depth consideration of ongoing changes in home-region politics is in many cases placed beyond the scope of study. However, while it is a demanding research agenda, studies can consider and compare populations defined by ethnic or national origins even while recognizing the multiple structures of identity that may overlap with these categories and engaging with people’s experiences of and orientations towards ongoing dynamics in the homeland (Basch et al., 2005; Abdi, 2015).

Trends suggested in this study are merely a starting point, and offer room for further analysis of correspondences and differences between Somali, Somalian, Ethiopian, and Somali-Ethiopian populations in multiple locations. Particularly critical in the current context of the Horn of Africa and the continuous emigration of youth from the region is how transformations in the Horn, which may be reflected in shifting diaspora identities abroad, may forge new patterns of political and economic involvement and connection that reshape future migration trajectories.

## Statistical data sources

Ethiopian Somali Regional State (ESRS), Ministry of Diaspora, spreadsheet list of diaspora returnees provided to author in June 2016, in author’s possession.

Statistics Canada (1996). Table 95F0182XDB. (All Statistics Canada data available from http://www.statcan.gc.ca/eng/subjects/index?MM=1).

Statistics Canada (2001). Table 97F0009XCB2001002.

Statistics Canada (2001). Table 97F0010xcb2001001.

Statistics Canada (2006). Table 97-557-XCB2006007.

Statistics Canada (2006). Table 97-562-XCB2006006.

Statistics Canada (2011). Table 99-010-X2011028.

Statistics Canada (2011). Table 99-010-X2011026.

Statistics Canada (2016). Table 98-400-X2016184.

Statistics Canada (2016). Table 98-400-X2016187.

Statistics Canada (2016). Table 98-400-X2016202.

United Nations, Department of Economic and Social Affairs (UNDESA) (2015). Trends in International Migrant Stock: Migrants by Destination and Origin (United Nations database, POP/DB/MIG/Stock/Rev.2015, available from http://www.un.org/en/development/desa/population/migration/data/estimates2/estimates15.shtml).

## Abbreviations

ACS: American Communities Survey
CD: Census Division
CMA: Census Metropolitan Area
CSD: Census Subdivision
ONLF: Ogaden National Liberation Front
UN: United Nations
UNDESA: United Nations Department of Economic and Social Affairs

## Footnotes

1 See ‘Massive anti-government protests signal Ethiopia’s deepening crisis.’ OPride, 11 October 2017. https://www.opride.com/2017/10/11/massive-anti-government-protests-signal-ethiopias-deepening-crisis/

2 Significant populations of Somalis also live in other areas of Kenya, including Nairobi.

3 Two prominent attacks by Somali al-Shabaab militants in recent years targeted Westgate Mall in Nairobi in September 2013 and Garissa University in April 2015.

4 Among many Somali youth, risking one’s life to migrate in search of a better life (an activity encapsulated as tacabir in Somali) is a respected endeavour, and (illegal) migration in search of asylum is common. A recent Somali radio program interviewed a group of Somalis who narrated how they had journeyed three months to reach northern Mexico in hopes of crossing the border into the US (‘Seddex bilood Ayaan tahriib kusoo jirnay’ [‘We were in transit for three months’] SBS Radio Somali service, Feb. 13, 2017).

5 For English-language discussion of these dynamics, see the Somalia Online forum, https://www.somaliaonline.com/community/topic/81028-tensions-rise-as-somali-region-as-illey-dispatches-delegation-to-the-us/

6 This dataset represents only a small sample of total diaspora return, but most records of returnees are not digitized.

## References

[CR1] Abdi CM (2015). Elusive jannah: the Somali diaspora and a borderless Muslim identity.

[CR2] Ali-Ali N, Koser K (2002). New approaches to migration? Transnational communities and the transformation of home.

[CR3] Barth F, Barth F (1998). Introduction. Ethnic groups and boundaries: the social organization of culture difference.

[CR4] Basch, L., Glick Schiller, N., & Szanton Blanc, C. (2005). *Nations unbound: transnational projects, postcolonial predicaments and deterritorialized nation-states *(Taylor & Francis e-Library). London and New York: Routledge.

[CR5] Brah, A. (2005). *Cartographies of diaspora: contesting identities*, (Taylor & Francis e-Library). London and New York: Routledge.

[CR6] Bulcha M (2002). The making of the Oromo diaspora: a historical sociology of forced migration.

[CR7] Byrne D (1977). The 1930 “Arab riot” in South Shields: a race riot that never was. Race & Class.

[CR8] Carrier N, Lochery E (2013). Missing states? Somali trade networks and the Eastleigh transformation. Journal of Eastern African Studies.

[CR9] Chacko E (2003). Ethiopian ethos and the making of ethnic places in the Washington metropolitan area. Journal of Cultural Geography.

[CR10] Danso R (2001). From “there” to “here”: an investigation of the initial settlement experiences of Ethiopian and Somali refugees in Toronto. GeoJournal.

[CR11] Gill N (2010). New state-theoretic approaches to asylum and refugee geographies. Progress in Human Geography.

[CR12] Grant R, Thompson D (2015). City on edge: immigrant businesses and the right to urban space in inner-city Johannesburg. Urban Geography.

[CR13] Habecker S (2012). Not black, but Habasha: Ethiopian and Eritrean immigrants in American society. Ethnic and Racial Studies.

[CR14] Hagmann T, Khalif MH (2006). State and politics in Ethiopia’s Somali region since 1991. Bildhaan: An International Journal of Somali Studies.

[CR15] Hammond L, Åkesson L, Eriksson Baaz M (2015). Diaspora returnees to Somaliland: heroes of development or job-stealing scoundrels?. Africa’s return migrants: the new developers?.

[CR16] Hansen P, Hammar A (2014). Diaspora returnees in Somaliland’s displacement economy. Displacement economies in Africa: paradoxes of crisis and creativity.

[CR17] Horst C, Kusow AM, Bjork SR (2007). The Somali diaspora in Minneapolis: expectations and realities. From Mogadishu to Dixon: the Somali diaspora in a global context.

[CR18] Jalata A (2001). Fighting against the injustice of the state and globalization: comparing the African American and Oromo movements.

[CR19] Jalata A (2002). The place of the Oromo diaspora in the Oromo National Movement: lessons from the agency of the “old” African diaspora in the United States. Northeast African Studies.

[CR20] Kelly JD, Kaplan M (2001). Represented communities: Fiji and world decolonization.

[CR21] King R, Christou A (2011). Of counter-diaspora and reverse transnationalism: return mobilities to and from the ancestral homeland. Mobilities.

[CR22] Kusow AM (2006). Migration and racial formations among Somali immigrants in North America. Journal of Ethnic and Migration Studies.

[CR23] Malkki LH (1992). National geographic: the rooting of peoples and the territorialization of national identity among scholars and refugees. Cultural Anthropology.

[CR24] Plaza S, Ratha D (2011). Diaspora for development in Africa.

[CR25] Povrzanovic Frykman M, Ali-Ali N, Koser K (2002). Homeland lost and gained: Croatian diaspora and refugees in Sweden. New approaches to migration: transnational communities and the transformation of home.

[CR26] Quayson A, Daswani G (2013). A companion to diaspora and transnationalism.

[CR27] Samatar AI, Bereketeab R (2013). The production of Somali conflict and the role of internal and external actors. The Horn of Africa: intra-state and inter-state conflicts and security.

[CR28] Schlenzka N (2009). The Ethiopian diaspora in Germany: its contribution to development in Ethiopia.

[CR29] Thompson, D. K. (2017a). Scaling statelessness: absent, present, former and liminal states of Somali experience in South Africa. *PoLAR: Political and Legal Anthropology Review*, *40*(1), 86–103.

[CR30] Thompson, D. K. (2017b). Visible and invisible diasporas: Ethiopian Somalis in the diaspora scene. Bildhaan: an International Journal of Somali Studies, 17, 1–31.

[CR31] Warnecke A (2010). Diasporas and peace: a comparative assessment of Somali and Ethiopian communities in Europe.

[CR32] Wettergren Å, Wikström H (2014). Who is a refugee? Political subjectivity and the categorisation of Somali asylum seekers in Sweden. Journal of Ethnic and Migration Studies.

